# Targeted Phagocytosis Induction for Cancer Immunotherapy via Bispecific MerTK-Engaging Antibodies

**DOI:** 10.3390/ijms232415673

**Published:** 2022-12-10

**Authors:** Stefania C. Carrara, Jan P. Bogen, David Fiebig, Julius Grzeschik, Björn Hock, Harald Kolmar

**Affiliations:** 1 Institute for Organic Chemistry and Biochemistry, Technical University of Darmstadt, Alarich-Weiss-Strasse 4, D-64287 Darmstadt, Germany; 2 Ferring Darmstadt Laboratories, Alarich-Weiss-Strasse 4, D-64287 Darmstadt, Germany; 3 Ferring Biologics Innovation Centre, Biopôle, CH-1066 Epalinges, Switzerland; 4 Aerium Therapeutics, Biopôle, CH-1066 Epalinges, Switzerland; 5 Centre for Synthetic Biology, Technical University of Darmstadt, Alarich-Weiss-Strasse 4, D-64287 Darmstadt, Germany

**Keywords:** macrophages, targeted phagocytosis, MerTK, EGFR, bispecific antibody, BiME

## Abstract

The Tyro, Axl, and MerTK receptors (TAMRs) play a significant role in the clearance of apoptotic cells. In this work, the spotlight was set on MerTK, as it is one of the prominent TAMRs expressed on the surface of macrophages and dendritic cells. MerTK-specific antibodies were previously isolated from a transgenic rat-derived immune library with suitable biophysical properties. Further characterisation resulted in an agonistic MerTK antibody that led to phospho AKT activation in a dose-dependent manner. In this proof-of-concept study, a MerTK-specific antibody, MerK28, was combined with tandem, biparatopic EGFR-binding VHH camelid antibody domains (7D9G) in different architectures to generate bispecific antibodies with the capacity to bind EGFR and MerTK simultaneously. The bispecific molecules exhibited appropriate binding properties with regard to both targets in their soluble forms as well as to cells, which resulted in the engagement of macrophage-like THP-1 cells with epidermoid carcinoma A431 cells. Furthermore, targeted phagocytosis in co-culture experiments was observed only with the bispecific variants and not the parental MerTK-binding antibody. This work paves the way for the generation of bispecific macrophage-engaging antibodies for targeted phagocytosis harnessing the immune-modulating roles of MerTK in immunotherapy.

## 1. Introduction

A specialised family of receptor tyrosine kinases is the Tyro3, Axl, and MerTK receptor (TAMR) family, which plays a number of crucial roles maintaining tissue homeostasis [1]. Structurally, the extracellular domains of all TAMRs are similar, being composed of two immunoglobulin-like domains that mediate ligand binding and two fibronectin type III repeats, with a conserved intracellular kinase domain, as with all other RTKs [2,3,4,5]. Two endogenous ligands, growth arrest-specific gene 6 (Gas6) [6] and anticoagulation factor protein S (Pros1) [7], bind the TAMRs, with Gas6 having a strong affinity for Axl but not for Tyro3 and MerTK, and Pros1 preferring Tyro3 and MerTK but not activating Axl [3,8]. Activation of the receptors is mediated by homo- and hetero-dimerisation of both the receptors and the ligands, resulting in TAMR-mediated signalling [5]. On macrophages, the most well-characterised signalling cascade is the phosphoinositide 3 kinase (PI3K)/AKT pathway, ultimately resulting in the phosphorylation of AKT (pAKT) through mediators in the signal transduction and altered gene expression [2,9,10].

Macrophages are commonly known as the professional phagocytes of apoptotic cells (AC), as they spend most of their time phagocyting apoptotic cells and fragments, with >10^9^ ACs generated in humans per day [3,11]. The maintenance of tissue homeostasis by efficient clearance of AC by macrophages is crucial to inhibit inflammatory and autoimmune responses against “self” antigens. MerTK is mainly expressed on myeloid cells pertaining to the immune system, including macrophages and dendritic cells (DC), and is involved in the clearance of ACs by recognising phosphatidylserine (PtdSer) exposed on ACs through bridging with its endogenous ligands, Gas6 and Pros1 [3,12,13,14,15]. Being largely absent on monocytes, MerTK becomes strongly upregulated upon monocyte-to-macrophage differentiation [2,16]. Furthermore, MerTK has been largely associated with “alternatively activated” M2 macrophages that aid in tissue homeostasis, wound healing, and fighting infections by secreting anti-inflammatory cytokines [2,17]. In line with their wound-healing immunosuppressive phenotype, M2-like macrophages have a greater capacity to clear ACs compared to the “classically activated” M1-like macrophages [2,18]. In vivo studies with mice containing a truncated version of MerTK showed reduced clearance of ACs, highlighting the importance of MerTK in this mechanism [13]. Additionally, a triple Tyro3/Axl/MerTK mice knock-out model showed normal development of the mice but exhibited elevated levels of pro-inflammatory cytokines (e.g., TNFα, IL-6). Furthermore, a diminished ability to clear ACs and increased auto-antibody production could be observed, which over time resulted in symptoms evocative of systemic lupus erythematosus, rheumatoid arthritis (RA), and psoriasis. A deeper analysis of single TAMR knock-outs exposed the role of MerTK in autoimmunity, contributing largely to the effects seen in the triple knock-out mice [19,20].

Nevertheless, MerTK may be seen as a double-edged sword, as it modulates the immune system by reducing immune cell activation through the clearance of ACs, but it has also been implied to have tumour-intrinsic functions due to its aberrant expression on several types of cancerous cells [21]. Targeting and inhibiting MerTK and other members of the TAMR family have been considered interesting anti-cancer strategies for reducing tumour development and promoting survival via the alleged MerTK-mediated signalling pathways, including the development of several pan-TAMR small molecule inhibitors and monoclonal antibodies [22,23,24,25,26,27]. 

However, evidence has emerged to cast doubt on TAMR blockage as a suitable anti-cancer therapy. As reported by Bosurgi et al., MerTK-deficient mice resulted in increased pro-inflammatory cytokine secretion and decreased AC clearance in colon cancer models, ultimately sponsoring a tumour-promoting tumour microenvironment (TME) [28,29]. Furthermore, on-target retinal toxicities were observed with treatment with an antagonistic MerTK antibody in cynomolgus monkeys, warranting the need for further investigation of MerTK-targeting therapy [22,29]. MerTK activity has also been shown to play a role in endothelial cell recruitment to the TME and to inhibit angiogenesis, thus hindering tumour development and growth [29,30].

To harness the increased expression of MerTK on tumour-associated macrophages (TAMs) in the TME, bispecific antibodies (bsAbs) were generated targeting a tumour-associated antigen (TAA) to generate bispecific macrophage engagers (BiMEs). While BiMEs have been previously described, they mediate macrophage engagement and activation by inhibiting the classical SIRPα and simultaneously targeting a TAA [31]. Along similar lines, CD47-directed bsAbs target the CD47/SIRPα axis by blocking CD47 signalling and restoring phagocytosis of tumour cells by macrophages [32]. In the hereby presented proof-of-concept study, targeted phagocytosis for cancer immunotherapy was substituted by targeting MerTK on macrophages and the epidermal growth factor receptor (EGFR) that is overexpressed on a plethora of tumour types [33,34,35]. By harnessing MerTK’s expression on phagocytes, this could ameliorate the immunosuppressive environment in the TME and result in targeted phagocytosis of malignant cells. This is, to the best of our knowledge, the first MerTK-mediated macrophage engager for immunotherapy.

## 2. Results

### 2.1. Characterisation of MerTK Monoclonal Antibody

As previously reported [36], an OmniRat-based immunisation campaign was initiated by genetic immunisation with the extracellular domain of MerTK. After fluorescence-activated cell sorting (FACS) of a Fab-library by yeast surface display (YSD), a panel of antibodies (mAbs) were isolated with favourable biophysical properties and high affinity binding to soluble human MerTK (hMerTK). Ten antibody candidates were reformatted as full-length IgG1 molecules, expressed via transient transfection in Expi293-F cells and purified by protein A chromatography. To further characterise these antibodies, all variants were initially tested for binding to the MerTK^+^ Jurkat cell line (Figure S1a) and to HEK293 cells (Figure S1b). While several antibody candidates showed decent binding to both cell lines, MerK17 and MerK28 were chosen for further characterisation as they displayed the highest binding in flow cytometric cell binding assays.

Their effect on the modulation of MerTK’s signalling transduction cascade was measured, as this is an important mechanism of action for the differentiation of macrophages and clearance of apoptotic cells. To this end, MerTK^+^ A431 cells were incubated with mAbs overnight, and subsequently the activation of pAKT was measured by homogenous time-resolved fluorescence (HTRF). While no activation was observed in the presence of MerK17, moderate pAKT activation was noted for MerK28, indicating agonistic properties of this antibody (Figure S1c). The most suitable stimulation time was investigated by a time-course on A431 and HEK293 cells, resulting in the strongest pAKT activation after 30 min with relative fold changes of 2.5 and 4.5 compared to the unstimulated control for A431 and HEK293 cells, respectively (Figure S1d,e). With the optimal stimulation time of 30 min, the HTRF pAKT assay was repeated on A431 cells to ensure MerK17 did not result in different activation profiles. Unsurprisingly, no stimulation was observed with MerK17, and dose-dependent stimulation was detected with MerK28 (Figure S1f).

**Figure 1:**
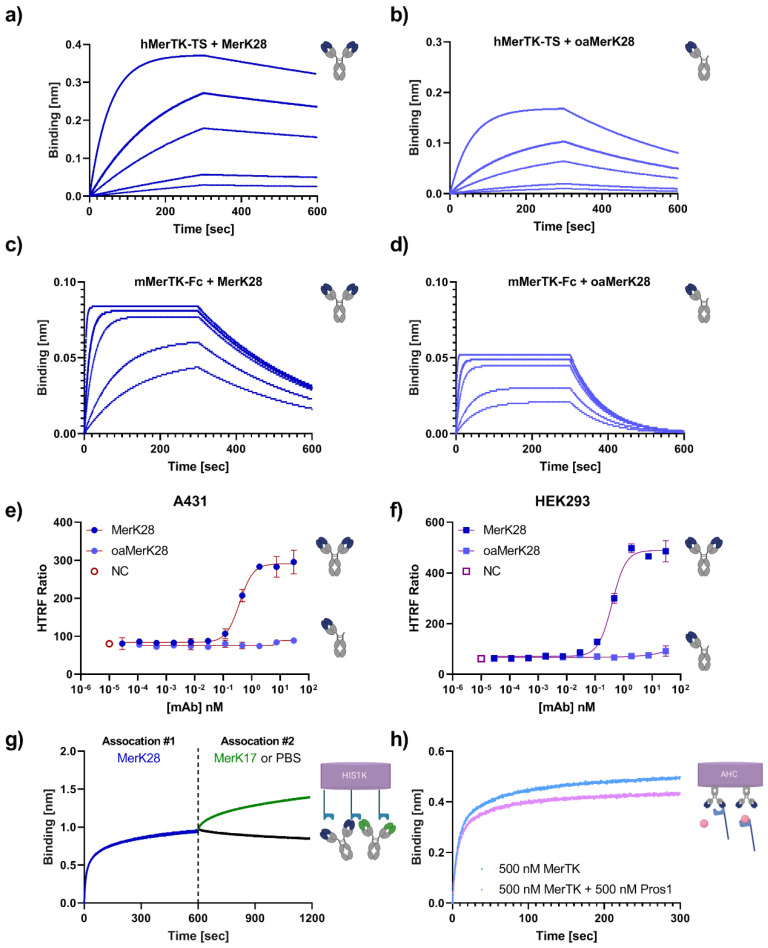
Characterisation of parental MerTK antibodies. Kinetics determination against soluble hMerTK-TS for either (**a**) MerK28 or (**b**) oaMerK28, and against mMerTK-Fc for (**c**) MerK28 or (**d**) oaMerK28. (**e**,**f**) Activation of signalling transduction with MerK28. pAKT HTRF assays with either A431 (**e**) or HEK293 (**f**) cells. Unstimulated cells are shown as “NC”, and the bivalent MerK28 and one-armed MerK28 (oaMerK28) are plotted. Error bars represent biological duplicate measurements. (**g**) Epitope binning of MerK28 (blue) and MerK17 (green) via BLI. Human MerTK-His_6_ was immobilised on HIS1K biosensors and associated to the indicated antibodies for 600 s each. PBS is shown in black. (**h**) Ligand competition assay for MerK28. Association to hMerTK (light blue) alone or to MerTK pre-incubated with Pros1 ligand (pink).

To further characterise the agonistic MerK28, its binding affinities to both hMerTK and murine MerTK (mMerTK) were determined by biolayer interferometry (BLI). A one-armed variant of MerK28, oaMerK28, was generated, and its MerTK-binding capability was also tested. Using in-house produced antigens, MerK28 and oaMerK28 were loaded onto AHC biosensors and associated to varying concentrations of either hMerTK-TS or mMerTK-Fc. Kinetics revealed low single-digit nanomolar affinity of MerK28 to hMerTK-TS (Figure 1a, Table 1) and a slight decrease in affinity with oaMerK28 (Figure 1b, Table 1). Cross-reactivity to mMerTK-Fc was revealed for both the bivalent and the one-armed MerK28 in the low-nanomolar range (Figure 1c,d). MerK17 did not exhibit cross-reactivity with murine MerTK. Dose-dependent activation of pAKT after mAb incubation was observed on both A431 (Figure 1e) and HEK293 (Figure 1f) cells for the bivalent MerK28, but not for the oaMerK28.

Due to the different effects on cell signalling of MerK17 and MerK28, an epitope binning via BLI was performed to confirm that different epitopes were being addressed. As seen in Figure 1g, MerK28 and MerK17 did not possess overlapping epitopes, as they were both able to bind to hMerTK-His_6_ simultaneously. These results were confirmed by reversing the order of association of the mAbs (Figure S2a). Additionally, it was investigated whether MerK28 competed for the ligand binding site of one of the MerTK ligands, Pros1. By pre-incubating MerTK with an equimolar amount of Pros1 and associating it to MerK28, a decreased layer thickness was observed over time compared to MerTK binding only, indicating that MerK28 competed for the Pros1 ligand binding site (Figure 1h). This effect was not noted with MerK17 (Figure S2b). These results further epitomise the agonistic characteristic of MerK28, as Pros1 naturally leads to receptor activation. Thus, MerK28 was chosen for further studies, as it allows the choice between an agonistic mAb in the bivalent form or binding in its monovalent form and is also cross-reactive to murine MerTK.

### 2.2. Generation and Characterisation of Bispecific MerTK/EGFR Variants

**Figure 2:**
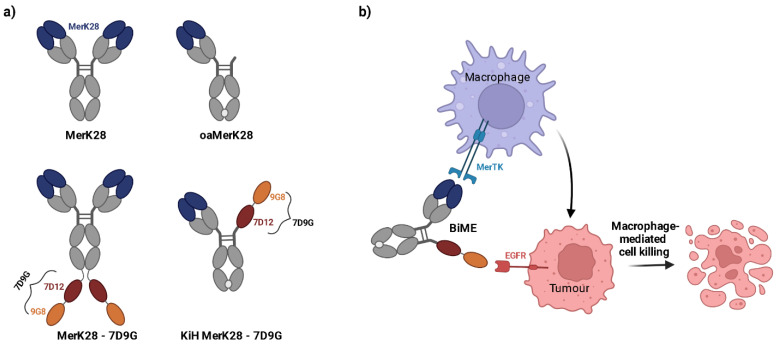
Bispecific MerTK/EGFR antibodies. (**a**) Architectures of the mono- and bispecific antibodies. VH and VL domains depicted in blue represent those for MerK28. The tandem VHHs together are termed 7D9G, with the red representing 7D12 and the orange 9G8 nanobodies. (**b**) Macrophage-tumour cells engagement with a bispecific macrophage engager (BiME). The blue cell represents macrophages expressing MerTK on their surface, whereas the pink cells represent tumour cells overexpressing EGFR.

After characterisation of the monovalent anti-MerTK antibody MerK28 and its one-armed counterpart oaMerK28, bispecific antibodies were generated by fusing tandem, biparatopic VHHs targeting EGFR (termed 7D9G), which were previously described elsewhere [37]. Two different architectures were chosen; one with the bivalent MerK28 antibody and a C-terminal fusion of 7D9G (hereafter termed MerK28-7D9G), and a bispecific with a single MerTK-binding moiety and only one 7D9G using the Knob-into-Hole technology (hereafter dubbed KiH MerK28-7D9G). All molecules and their respective architectures are depicted in Figure 2a. As the KiH MerK28-7D9G variant was equivalent to the oaMerK28 in terms of MerTK binding valency, the agonistic properties of the molecule were bypassed, allowing for a simple macrophage-engager instead of -activator (Figure 2b).

After transient production in Expi293-F cells and purification either via Protein A chromatography or a His/Strep purification for MerK28-7D9G or KiH MerK28-7D9G, respectively, SDS-PAGE gel analysis revealed the expected bands under both reducing and non-reducing conditions (Figure S3). For the MerK28-7D9G variant, additional bands were noticeable under non-reducing conditions, indicating decreased stability, likely due to the addition of two tandem VHHs with significant flexibility. Yields and thermal stability were within acceptable ranges for bispecific antibodies, with surprisingly higher yields for both bispecifics compared to the parental MerK28 clone (Table 1). Thermal stability investigation by SYPRO-Orange incorporation indicated exceptional stability with melting temperatures (T_M_) between 74.0–75.0 °C (Table 1). The high global T_M_s for the molecules suggest decent stability.

**Figure 3:**
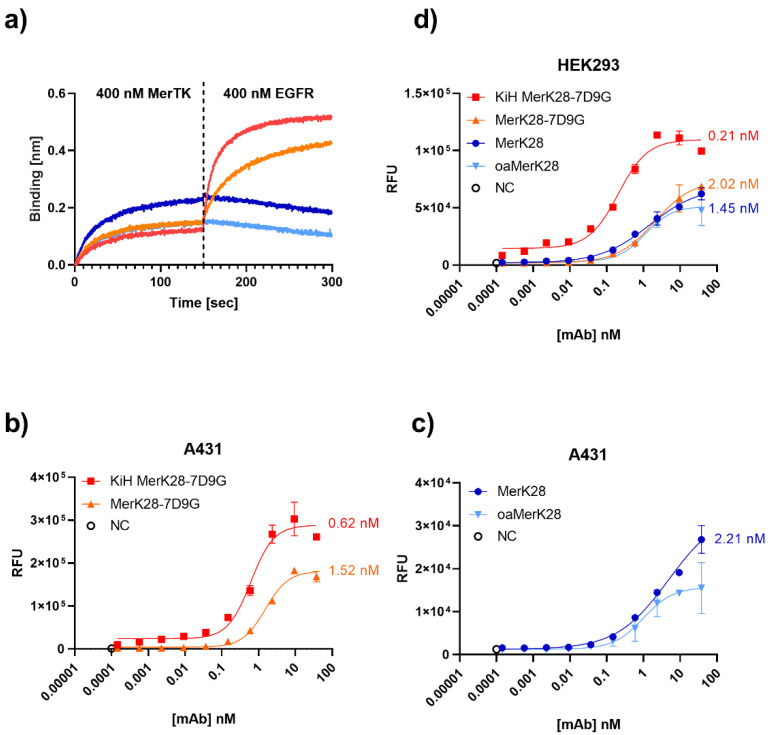
Bispecific MerTK/EGFR antibodies and their binding capabilities. (**a**) Simultaneous binding of hMerTK and EGFR via BLI. Antibodies were loaded onto AHC biosensors and then associated first to MerTK and then to EGFR for 300 s. The colour-coding is the same as represented in the legend for (**d**). Cell titration on (**b**,**c**) A431 and (**d**) HEK293 cell lines by staining the antibodies with anti-human Fc PE and plotting the relative fluorescence units (RFU) versus the antibody concentration. The negative controls incubated only with secondary antibodies are shown as “NC”. Error bars represent biological duplicates.

To ensure that the bispecific molecules could bind both targets simultaneously in the soluble form, a BLI-based assay was performed by first associating the antibodies to hMerTK and then to EGFR. As can be observed in Figure 3a, the bsAbs were able to bind both antigens. The same result was observed in the opposing order, verifying its simultaneous binding capacity (Figure S2c).

To determine an on-cell affinity, A431 and HEK293 cells were titrated. On A431 cells, which are EGFR^+++^/MerTK^+^, 620 pM and 1.52 nM on-cell binding affinities were determined for KiH MerK28-7D9G and MerK28-7D9G, respectively (Figure 3b). MerK28 had an on-cell affinity of 2.21 nM, even though the fold induction was significantly lower, as no EGFR binding was possible (Figure 3c). While HEK293 cells are also EGFR^+^, the lower expression compared to A431 cells was a more realistic model. The on-cell affinities on HEK293 cells remained in a similar range, with 0.21 nM and 2.02 nM for KiH MerK28-7D9G and MerK28-7D9G, respectively, and 1.45 nM for MerK28 (Figure 3d). The on-cell affinity was unexpectedly higher for the KiH bispecific compared to MerK28-7D9G, as the latter should have been able to bind both MerTK and EGFR more effectively due to having two and four binding moieties, respectively. An explanation for this could be the intermittent molecule flexibility not providing any advantage in this precise architecture compared to the KiH variant. Nevertheless, on-cell affinity in the single-digit to low nanomolar range was determined for the EGFRxMerTK bispecifics. Unspecific binding to other cell-surface proteins by the parental MerK28 antibody was ruled out by staining MerTK^-^ ExpiCHO cells (Figure S2d). For verification, affinity was determined by BLI for soluble EGFR and hMerTK (Figure S4, Table 1). Both bsAbs exhibited picomolar affinities towards EGFR and very similar K_D_s for hMerTK, comparable to the parental MerK28.

### 2.3. Influence of Bispecifics on EGFR Signalling Cascade

**Figure 4:**
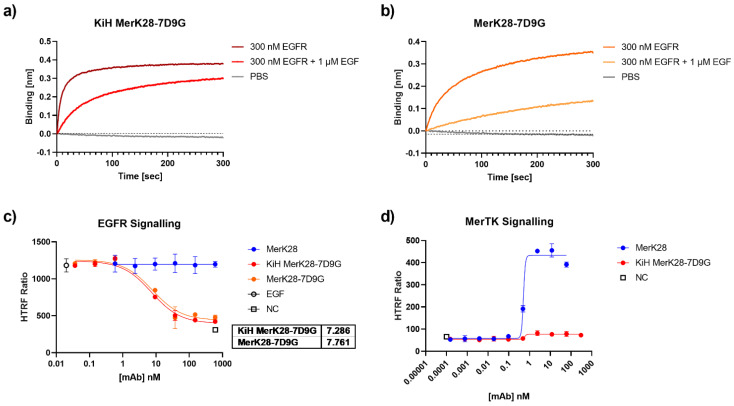
EGF competition and inhibition through 7D9G. EGF competition assay via BLI for (**a**) KiH MerK28-7D9G and (**b**) MerK28-7D9G. Antibodies were loaded onto AHC biosensors and either associated to 300 nM EGFR (dark red or orange), 300 nM EGF pre-incubated with 1 µM EGF (lighter red and orange), or only PBS (grey). (**c**) HTRF pAKT of A549 cells stimulated with 20 ng/mL EGF after pre-incubation with the indicated antibodies and respective concentrations. Negative control (NC) represents unstimulated cells, while EGF control shows maximum activation of pAKT. (**d**) Investigation of MerTK signalling pathway on HEK293 cells after 30 min incubation with either parental MerK28 (blue) or KiH MerK28-7D9G (red). Error bars represent the standard deviation of biological duplicates.

With the promising biophysical properties of the bispecific molecules, their ability to inhibit the EGF/EGFR signalling cascade was examined. An EGF competition assay was first performed via BLI by incubating EGFR with an excess of EGF and observing the ability of the antibodies to bind to the complex. For both KiH MerK28-7D9G and MerK28-7D9G, binding to EGF-competing epitopes could be observed (Figure 4a,b), suggesting that it could inhibit the EGF-mediated signalling transduction cascade of EGFR. The binding of the biparatopic VHH 7D9G to EGFR was mapped by using EGFR mutants displayed on yeast cells, as previously described [38]. By measuring the binding of KiH MerK28-7DG to the different YSD-displayed fragments by flow cytometry, binding to EGFR could be allocated to domain III, one of the EGF-binding domains on the receptor [39], confirming previous results [37] (Figure S5).

To verify these results in a cellular setting, A549 cells were pre-incubated with varying concentrations of antibodies for 1 h and subsequently stimulated with EGF for 10 min. Analysis of pAKT by HTRF resulted in IC_50_ values of 7.29 and 7.76 nM for KiH MerK28-7D9G and MerK28-7D9G, respectively (Figure 4c). Further, as the KiH bispecific variant should be equivalent to the one-armed MerK28 (oaMerK28) variant concerning its ability to activate the MerTK signalling pathway, or rather lack thereof, HEK293 cells were incubated with the bsAbs and downstream signalling by pAKT activation was measured. While the parental mAb MerK28 resulted in strong activation, the bispecific KiH MerK28-7D9G did not (Figure 4d). Thus, this construct could be used as a macrophage-engager without activating MerTK signalling.

### 2.4. Differentiation of THP-1 Cells from Monocytic (Mθ) to Macrophage-like (M0) State

To assess the phagocytic capabilities of macrophages, the THP-1 cell line first had to be differentiated into a macrophage-like state, hereafter termed M0, through the addition of phorbol 12-myristate-13-acetate (PMA). Cells were incubated with 20 ng/mL PMA for 24 h and specific markers were subsequently measured to ensure the successful differentiation of THP-1 cells into an M0 macrophage-like state. Initial gene expression data revealed over 15-fold and nearly 300-fold relative mRNA increase in *Itgam (Cd11b)* and *Il-1b* after 24 h PMA incubation (Figure S6a) and the expected change in morphology was observed (Figure S6b). To confirm the gene expression data, THP-1 cells were stained with anti-CD11b or anti-CD14 antibodies. Both markers showed a significant increase after PMA differentiation, with a 40-fold increase in cell-surface CD11b, and a nearly 4-fold increase in CD14 levels compared to Mθ THP-1s (Figure S6c,d). More importantly, members of the TAMR family were also analysed by gene expression, resulting in a significant increase in *MerTK*, as well as *Axl* (Figure S6e). Nevertheless, only a very modest increase in mRNA levels was observed for *Axl* compared to *MerTK*, which is in line with MerTK playing a more significant role on macrophages compared to its other TAMR members. Further, increased levels of *Cd274* (*Pd-l1*) were also observed (Figure S6e). The increase in *MerTK* mRNA levels was also corroborated by measuring the cell-surface levels by flow cytometry (Figure S6f). The significant increase at both gene and protein levels of such M0 markers and the very evident change in morphology confirmed the macrophage-like state of M0 PMA-primed THP-1 cells.

### 2.5. M1- and M2-Polarised Macrophage-like Characteristics of THP-1 Cells

**Figure 5:**
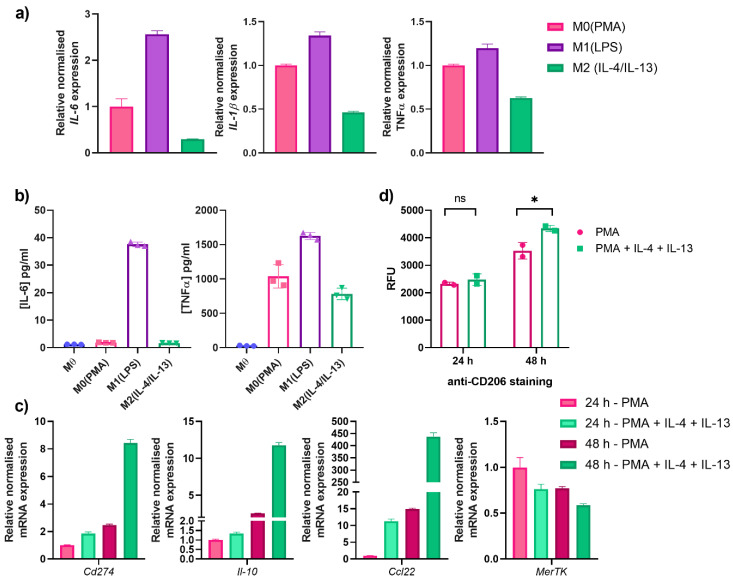
Differentiation of macrophage-like THP-1 cells to M1- and M2-state. (**a**) Gene expression analysis of THP-1 cells differentiation with PMA (M0—pink), LPS (M1—violet) or IL-4/IL-13 (M2—teal) for 24 h. The data are normalised to housekeeping genes and set relative to the M0(PMA) control. (**b**) Secretion of IL-6 and TNFα in cell culture supernatants as determined by ELISA. (**c**) Gene expression analysis of PMA- (M0—pink shades) and PMA/IL-4/IL-13-incubated cells (M2—greens shades) after either 24 h (lighter colours) or 48 h (darker shades) incubation. Analysis was performed by normalising the data to housekeeping genes and setting the values relative to the M0 24 h control. (**d**) CD206-cell surface staining of differentiated cells by flow cytometry. After differentiation, THP-1 cells were stained with anti-CD206 PE-conjugate and measured by flow cytometry. The mean relative fluorescence units (RFU) were determined and plotted for each individual duplicate. One-way ANOVA analysis performed using GraphPad Prism: ns: non-significant, * *p* value < 0.05.

While THP-1 cells do not represent the complexity of human monocyte-derived macrophages, they are widely used as a model system for in vitro studies. To this end, macrophage-like M0 THP-1 cells were further differentiated by external stimuli to an M1-like or M2-like differentiation state by the addition of lipopolysaccharide (LPS) or IL-4 and IL-13, respectively. Characterisation of M1-differentiated cells was performed by measuring *Il-6*, *Il-1β*, and *Tnfα* mRNA levels (Figure 5a). Compared to the M0 control, significant upregulation was observed for all three genes upon incubating THP-1 cells with LPS, but not with IL-4 and IL-13 as expected. Secretion of cytokines (IL-6 and TNFα) was determined by ELISA and were in line with the gene expression data (Figure 5b). For IL-6, an M1-specific increase was noted, whereas TNFα secretion was already increased after PMA incubation (M0), which was then increased further upon LPS treatment (Figure 5b).

As MerTK is involved in M2-differentiated macrophages, M0 THP-1 cells were differentiated to investigate its phagocytic capabilities. To this end, Mθ THP-1 cells were incubated with either PMA (M0) or PMA with IL-4 and IL-13 simultaneously (designated M2). M2 markers were investigated by comparing M0 and M2 macrophage-like THP-1 cells after 24 and 48 h incubation. By gene expression, two important M2 markers, *Il-10*, and *Ccl22*, were shown to increase upon the addition of IL-4 and IL-13 compared to only PMA incubation (Figure 5c). While only a modest increase was shown after 24 h, the relative normalised mRNA expression was drastically increased after 48 h incubation, with a 12-fold and a 450-fold increase for *Il-10* and *Ccl22*, respectively, relative to the 24 h M0 control (Figure 5c). 

In the peculiar case of *MerTK* levels, an unexpected, modest decrease was observed compared to the 24 h M0 control (Figure 5c). While this was not particularly expected, as literature reports upregulation of MerTK in an M2-state, an explanation for this could be the increase in metalloproteinases, such as a disintegrin and metalloproteinase 17 (ADAM17), which is known to cause MerTK shedding and could thus have an influence on its expression levels [40]. Furthermore, the time-dependent decrease in the PMA M0 control from 24 to 48 h also substantiates this supposition. RT-qPCR revealed an 8-fold increase in relative *Cd274* mRNA levels—another marker of MerTK-dependent M2 differentiation—after 48 h incubation with IL-4 and IL-13 compared to the 24 h M0 control (Figure 5c). A further important M2-specific marker is CD206, which is known to not be expressed on M1 macrophages [41]. Staining of differentiated THP-1 cells with an anti-CD206 antibody revealed no significant difference between PMA- (M0) and PMA/IL-4/IL-13 (M2)-differentiated THP-1 cells after 24 h incubation (Figure 5d). Nonetheless, a longer incubation time of 48 h revealed a statistically significant increase in CD206 surface levels in M2-differentiated macrophage-like cells (Figure 5d). The increase in M1- and M2-specific markers confirmed the successful THP-1 differentiation to the respective macrophage-like states.

### 2.6. Phagocytosis of Anti-MerTK Parental Antibodies and Anti-MerTK/EGFR Bispecifics

**Figure 6:**
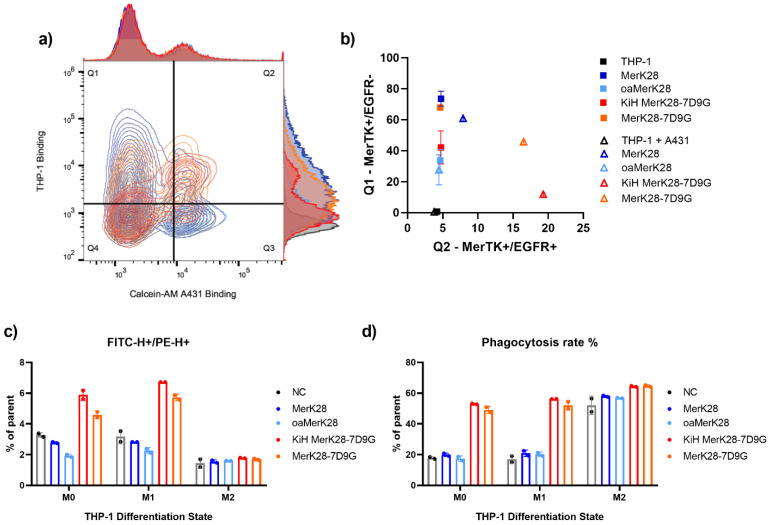
Macrophage-engagement and phagocytosis of tumour cells. (**a**) Contour plots of A431 binding on the x-axis (FITC fluorescence) and THP-1 binding on the y-axis (PE fluorescence) following the colour-coding of (**b**). Double-positive events in the upper-right Q2 quadrant show simultaneous engagement of THP-1 and A431 cells, whereas the upper-left Q1 quadrant represents MerTK-binding only. (**b**) Frequency of parent of Q1 and Q2 from (**a**). Coloured squares represent incubation of antibodies only on THP-1 cells, whereas the triangles represent co-incubation of pre-stained THP-1 cells and Calcein-AM-stained A431 cells. (**c**) Quantitation of CD14+-THP-1 cells and Calcein-AM red—orange stained A431 cells for phagocytosis assays. The negative control (NC) represents co-incubation of both cell types without antibody treatment. (**d**) Determination of phagocytosis rate (%) after co-incubation of THP-1 cells in different differentiation states together with A431 cells. Error bars represent the standard deviation of at least biological duplicates.

The simultaneous binding of macrophages and tumour cells is crucial for a macrophage-engaging bispecific antibody. To test this in a cellular context, THP-1 cells were pre-incubated with the respective antibodies and then stained with a PE-conjugated secondary antibody. Calcein-AM-stained A431 cells were added to the stained THP-1 cells and simultaneous MerTK-binding on THP-1 and EGFR-binding on A431 cells was measured using a flow cytometer. The MerK28 and oaMerK28 antibodies only displayed THP-1 binding in both monoculture and co-culture with A431 cells (Figure 6a,b). The quantification of their binding compared to the negative control resulted in 80% and 35% binding for MerK28 and oaMerK28, respectively, which corroborates with their MerTK-binding valency (Figure 6a,b). After co-incubation of pre-stained THP-1 cells and Calcein-AM-stained A431 cells, double positive events were only observed for the bispecific molecules (Figure 6a,b), as they are capable of binding both cell types. The quantitation of THP-1-only binding resulted in similar frequencies compared to the respective monospecific control (MerK28 for MerK28-7D9G, or oaMerK28 for KiH MerK28-7D9G). As MerK28-7D9G has two sets of tandem VHHs fused C-terminally, its EGFR-binding resulted in a higher engagement compared to the KiH variant (Figure 6b).

Furthermore, the ability of targeted phagocytosis with pre-characterised THP-1 macrophage-like states (M0, M1, or M2) was tested via flow cytometric analysis after co-incubation of the differentiated THP-1 cells with the respective antibody variants and Calcein-AM red—orange stained A431 cells. The entire complex after co-incubation was stained with an anti-CD14-FITC conjugated antibody to be able to gate the THP-1 cells. For both M0 and M1-like THP-1 cells, treatment with the bispecific antibodies led to a significant increase in double-positive events (Figure 6c and Figures S7 and S8). In the case of M2 macrophages, the cell-surface CD14 levels were very low, and thus no significant differences were observed. These double-positive events were gated further to determine the phagocytosis rate, as exemplified in Figure S9 for M1-like macrophages.

Quantitation of phagocytosis resulted in a significant increase for all differentiation states with both bispecific variants compared to the negative control, and non-significant changes upon incubation of the cells with MerTK-binding antibodies only (Figure 6d). In the case of M2-differentiated THP-1 cells, higher basal phagocytosis was observed, and moderate differences were noted with the bsAbs (Figure 6d). Thus, targeted phagocytosis with EGFR^+^ tumour cells was seen for all macrophage-like states in vitro.

## 3. Discussion

In this proof-of-concept study, bispecific macrophage-engaging antibodies were generated by combining MerTK-binding Fabs with tandem, biparatopic EGFR-targeting VHHs in different architectures. Contrary to MerK17, MerK28 exhibited cross-specific binding to human and murine MerTK, and was thus considered further. The parental MerTK clone, MerK28, exhibited agonistic properties in the bivalent form, losing its ability to activate MerTK-dependent signalling transduction when a one-armed variant was generated. Thus, the two bispecific antibodies had either mono- or bivalent MerTK binding to mediate the activation of MerTK. As activation of MerTK is said to lead to an immunosuppressive environment, the KiH MerK28-7D9G variant would be a more suitable fit for immunotherapy, as its capacity to activate pAKT through MerTK signalling was abolished. Further, no robust advantages for bivalent MerTK-binding were observed throughout this study when comparing both bispecific molecules. Due to the flexible nature of VHHs, and especially tandem VHHs, it may not be suitable to combine a total of four VHHs to the C-terminus of the Fc fragment, as the resulting molecule could have very flexible termini and could therefore be relatively unstable.

Characterisation of the 7D9G tandem VHHs resulted in picomolar affinity to EGFR as reported previously [37], inhibition of EGF binding to its receptor, and inhibition of EGF-mediated signal transduction through pAKT. The binding of 7D9G was mapped to domain III of the extracellular EGFR, which is involved in EGF binding [39], confirming its mechanism of action. By combining a potent EGFR binder with a medium-affine MerTK binder, the bispecific molecules will be more likely to reach the tumour microenvironment, where MerTK binding can take place to bridge tumour-associated and tumour-infiltrating macrophages with tumour cells and lead to phagocytosis in a targeted manner. In this context, no MerTK activation is required, and might even lead to counterproductive repercussions, as previously reported by Waterborg and colleagues [42]. For M0-like and M1-like THP-1 cells, targeted engagement and phagocytosis were observed with the bispecific antibodies after co-incubation with epidermoid carcinoma cells overexpressing EGFR. On the other hand, M2-differentiated THP-1 cells resulted in higher basal phagocytosis, as expected. Differences in phagocytosis with the bsAbs compared to the parental mAb were observed, however they were not as large as in the other differentiation states. An explanation for this could be the protease-mediated cleavage of MerTK, compromising the ability of the antibodies to bind to the THP-1 cells.

Proteolytic cleavage via ADAM17 results in soluble forms of MerTK that compete with surface-bound MerTK and inhibits efferocytosis [43]. A possible advantage of a bispecific antibody with bivalent agonism to MerTK might be in the recruitment of soluble MerTK after ADAM17 cleavage and shedding to restore TAMR-dependent efferocytosis; however, this requires further investigation. Further experiments with macrophages in conjunction with an appropriate mouse model will reveal the potential of bispecific MerTKxEGFR antibodies for cancer immunotherapy. Additionally, a thorough analysis of the role of these bispecific molecules and their activation of the MerTK pathway in the clearance of apoptotic cells should be performed, as the clearance of apoptotic cells within the TME is largely hindered by chronic inflammation. Effective clearance of ACs may have the potential to ameliorate the immunosuppressive environment and thus reduce anti-inflammatory effects from non-efficiently cleared ACs. Furthermore, combination therapy or the addition of PD-L1-binding moieties to the bispecific antibodies generated herein could enhance tumour cell-killing and avoid the upregulation of PD-L1 by MerTK in a potentially immunosuppressed TME. Taken together, harnessing MerTK expression on tumour-infiltrating macrophages via bispecific antibodies binding to tumour-specific markers might pave the way for interesting therapeutic strategies resulting in the targeted phagocytosis of tumour cells. 

## 4. Materials and Methods

### 4.1. Cell Culture

The following cell lines were purchased from DSMZ (Brunswick, Germany): human epidermoid carcinoma A431 (ACC 91), human embryonic kidney HEK293 (ACC 305), human lung carcinoma A549 (ACC 107), and human acute monocytic leukaemia THP-1 (ACC 16). Cell maintenance and production of soluble proteins were performed as previously described [44,45]. The cancer cell lines were chosen based on their EGFR overexpression in the case of A431 and A549, and the endogenous expression of MerTK on HEK293 cells.

Monocytic THP-1 cells (Mθ) were differentiated into macrophage-like cells (M0) by incubating with 20 ng/mL phorbol 13-myristate 13-acetate (PMA, Sigma Aldrich, Taufkirchen, Germany) for 24 or 48 h. Afterwards, the medium was exchanged and the M0 THP-1 cells were incubated in growth medium or differentiated further. Differentiation into an M1-like state was achieved by incubating with 250 ng/mL lipopolysaccharides (LPS) for 24 h. For an M2-like state, THP-1 cells were differentiated with 20 ng/mL PMA, 20 ng/mL IL-4 and 20 ng/mL IL-13 for 48 h.

### 4.2. Gene Expression Analysis for THP-1 Differentiation

RNA isolation was performed after lysis of cells using the RNeasy Mini Kit (QIAGEN, Hilden, Germany). Pure RNA was ensured by determining the concentration and ensuring an absorbance ratio (A_260/280_) of 2.0. For RT-qPCR, iTaq Universal SYBR Green One-Step kit (Bio-Rad, Feldkirchen, Germany) was used, primers for *Gapdh, Hprt1, MerTK,* and *Axl* were ordered from IDT (Coralville, IA, USA), while the rest were ordered from Sigma Aldrich (Sequences in Table S1). RT-qPCR and subsequent gene expression analysis were performed using a CFX Connect instrument (Bio-Rad) with the integrated CFX Manager software. Normalised relative mRNA expression was determined by normalising the data to the housekeeping genes (*Gapdh* and *Hprt1*) and setting gene expression relative to the Mθ THP-1 sample.

### 4.3. Cloning and Production of Antibodies

The generation of anti-MerTK antibody clone MerK28 was described previously [36]. For the generation of bispecific antibodies, two tandem, biparatopic VHHs (termed 7D9G) binding EGFR were chosen [37]. Cloning of the bispecific variants was performed using Golden Gate Cloning. Primers with *Sap*I overhangs were designed and ordered at Sigma Aldrich. For MerK28-7D9G, 7D9G was added to the C-terminus of the Fc region. For the KiH MerK28-7D9G, the MerK28 VH region was re-cloned into a plasmid containing a CH1 domain and a Knob-mutated Fc fragment, and the 7D9G was added to a Hole-mutated Fc fragment (lacking a CH1 region and containing a partial hinge). The knob-into-hole (KiH) technology was used to ensure heterodimerisation of the Fc fragments. Cloning was performed using Q5 DNA Polymerase (New England Biolabs, Frankfurt am Main, Germany), and Golden Gate cloning was performed with *Sap*I restriction enzyme (New England Biolabs).

For production and purification of the antibodies, Expi293-F cells were transfected as described above. Five days post-transfection, the cells were harvested by centrifugation and the supernatant was sterile-filtered. MerK28 and MerK28-7D9G were subsequently purified using Protein A chromatography followed by a desalting step into PBS. The one-armed MerK28 (oaMerK28) and the KiH bispecific variant were purified by a two-step purification through His_6_- and StrepII-tags to ensure heterodimers, as previously described [45].

### 4.4. Affinity Determination, EGF Competition and Epitope Binning with Biolayer Interferometry (BLI)

For affinity determination, 10 µg/mL of the respective antibody was loaded onto anti-human Fc capture biosensors (AHC, Sartorius, Göttingen, Germany) until a threshold of 0.8 nm was reached. Different antigen concentrations were associated to the antibodies for 300 s, followed by a dissociation step for 300 s in Kinetics Buffer (KB). For soluble human MerTK and EGFR, a range of 200 nM–0 nM was measured using in-house produced antigens (hMerTK-TS, His-EGFR-TS). Murine MerTK as an Fc-fusion was used in a concentration range of 300–0 nM for MerK28.

EGF competition, epitope binning, and simultaneous binding assays were performed as previously described [45]. In brief, EGF competition was performed by pre-incubating 300 nM His-EGFR-TS (in-house produced) with 1 µM EGF and associating the loaded antibodies for 300 s. Similarly, Pros1 competition was performed with 500 nM hMerTK-TS (produced in-house) and equimolar amounts of Pros1 (R&D Systems, Wiesbaden, Germany). Epitope binning was performed by loading hMerTK-His_6_ onto anti-HIS1K biosensors (Sartorius, Göttingen, Germany). MerK28 was first associated to hMerTK for 600 s, followed by associating to MerK17 or PBS afterwards. Lastly, simultaneous binding was performed by loading antibodies onto AHC biosensors, and similarly to the epitope binning, sequentially associating to different antigens.

### 4.5. Phospho AKT Signalling Assays

To investigate either MerTK- or EGFR-signal transduction pathways, 50,000 or 200,000 cells/well were seeded onto either 96-well or 24-well plates for HTRF and Western blot analysis, respectively, in complete growth medium, and incubated at 37 °C in 5% CO_2_ until cells became adherent. Thereafter, cells were serum-starved for 12–18 h in growth medium deprived of FBS. For MerTK activation, serum-starved cells were incubated with the indicated antibodies for 30 min. For EGFR-signalling, the cells were pre-treated with antibodies for 1 h and subsequently stimulated with 20 ng/mL EGF for 10 min. After stimulation, the cells were lysed in either 30 or 80 µL/well lysis buffer for 96- and 24-well plates, respectively. The lysis buffer for subsequent western blot analysis was composed of 1x Tris MSD lysis buffer supplemented with 1x PhosSTOP (Roche, Basel, Switzerland) and 1x Halt Protease and Phosphatase Inhibitor cocktail (Thermofisher Scientific, Erlangen, Germany). For the phospho AKT HTRF kit (64AKSPEG, Cisbio/Perkin Elmer, Codolet, France), the lysis buffer was prepared according to the manufacturer’s instructions.

Western blot analysis was performed by loading 15 µL sample onto a 4%–15% Mini-PROTEAN TGX precast gel (Bio-Rad) in reducing Laemmli buffer. After separation by SDS PAGE, the Trans-Blot Turbo Transfer System (Bio-Rad) was used to transfer the proteins onto a nitrocellulose membrane. The membranes were initially blocked in TBS-T (TBS containing 0.1% Tween-20) with 5% bovine serum albumin (BSA) for 1 h at RT. After 3 wash steps with TBS-T, the membranes were incubated with primary antibody diluted in TBS-T for 1 h. Washing was repeated three times and incubated with AP-conjugated secondary antibody in TBS-T for a further hour. After the final washing steps, 5 mL 1-step NBT/BCIP substrate solution (Thermofisher Scientific) was added per membrane and incubated until colour was developed. In order to stop the reaction, the membranes were washed thoroughly with water. Antibodies used: phospho AKT (Ser473) (#4060, Cell Signaling Technology, Frankfurt am Main, Germany), total AKT (#9272, Cell Signaling Technology).

### 4.6. Cell Titration, Macrophage Markers and Simultaneous Binding by Flow Cytometry

To determine an on-cell EC_50_, target cells were washed with ice-cold PBS-F (PBS containing 2% FBS) and incubated with the desired antibody concentration diluted in PBS-F for 30 min on ice in a 96-well U-bottom plate. After a washing step, the pelleted cells were incubated with anti-human Fc-PE conjugated antibody (1:80 dilution, Thermofisher Scientific) for 15 min on ice. Washing with ice-cold PBS-F was performed three times before measuring on a CytoFlex S (Beckman Coulter, Krefeld, Germany) flow cytometer. Cells stained only with secondary antibody were used as a negative control and in order to adjust the parameters. As a negative cell line, ExpiCHO-S (Thermofisher Scientific) were stained in the same way.

Simultaneous binding of M0 THP-1 cells and the A431 tumour cells was performed by pre-incubating THP-1 cells with the desired antibody concentration and stained with anti-human Fc-PE as described above. A431 cells were stained with 1 µM Calcein-AM according to the manufacturer’s protocol and subsequently co-incubated with pre-stained THP-1 cells. After 30 min, duplets were analysed by flow cytometry.

Cell surface expression of macrophage markers were measured by flow cytometry using the same procedure as above. Antibodies used: mouse anti-human CD11b-PE (#12-0118-42, Thermofisher Scientific), mouse anti-human CD14-FITC (#11-0149-42, Thermofisher Scientific), and mouse anti-CD206 PE-conjugate (Clone 19.2, Thermofisher Scientific). Data visualisation was performed using FlowJo v10 software (BD Biosciences, Franklin Lakes, NJ, USA) and GraphPad Prism 8.0. (San Diego, CA, USA).

### 4.7. Phagocytosis

To determine the rate of phagocytosis, differentiated THP-1 cells were trypsinised and washed thoroughly before incubating with the desired antibody concentration for 1 h at 37 °C. Simultaneously, target cells were trypsinised, washed, and stained with 5 µM Calcein-AM red–orange for 1 h at 37 °C. Subsequently, both cell types were mixed in a 1:5 effector-to-target ratio and incubated further. Then, the mixture was washed three times with ice-cold PBS and stained with anti-CD14-FITC for 20 min on ice. A final wash series was performed, and then the cells were analysed via flow cytometry on a CytoFlex S cytometer. Macrophages were gated through their CD14^+^ expression, and then the number of CD14^+^/Calcein-AM red–orange events were considered as phagocytosed A431 cells by the macrophages. The rate of phagocytosis was determined by the frequency of CD14^+^/Calcein-AM red–orange^+^ as a percentage of the parent population.

## Figures and Tables

**Table 1:** Biophysical properties of MerK28 and its derived bispecifics. Abbreviations: TS—TwinStrepII-Tag, n.d.—not determined.

Variant	Affinity Determination			Yield for 1 L (mg)	Thermal Stability (°C)
	hMerTK-TS (nM)	mMerTK-Fc (nM)	EGFR (pM)		
**MerK28**	26.9 ± 0.238	2.31 ± 0.0845	-	67.8	75.0
**oaMerK28**	30.5 ± 0.499	4.83 ± 0.205	-	56.2	74.0
**MerK28-7D9G**	34.0 ± 0.271	n.d.	155 ± 68.3	90.1	75.0
**KiH MerK28-7D9G**	34.7 ± 0.307	n.d.	389 ± 22.0	154.0	76.0

## Data Availability

Data are contained within the article or supplementary material.

## Supplementary Materials

The supporting information can be downloaded at: https://www.mdpi.com/article/10.3390/ijms232415673/s1.
