# Supplementary material for: Targeted Phagocytosis Induction for Cancer Immunotherapy via Bispecific MerTK-Engaging Antibodies
Source: Int J Mol Sci. 2022 Dec 10;23(24):15673. doi: 10.3390/ijms232415673 (PMC9779728; doi:10.3390/ijms232415673)
Supplement: Supplementary file 1 [file ijms-23-15673-s001.zip › ijms-2035423-supplementary.pdf]

Supplementary Information

Targeted phagocytosis induction for cancer immunotherapy *via* bispecific MerTK-engaging antibodies

Stefania C. Carrara [1,2], Jan P. Bogen [1,2], David Fiebig [1,2], Julius Grzeschik [3], Björn Hock [4] and Harald Kolmar [1,5,*]

Table S1: RT-qPCR SYBR Green primers

**Gene Name**	**Forward**	**Reverse**
Gapdh	ACATCGCTCAGACACCATG	TGTAGTTGAGGTCAATGAAGGG
Hprt1	TTGTTGTAGGATATGCCCTTGA	GCGATGTCAATAGGACTCCAG
MerTK	CTGTGTTTCTGAATGAATCTAGTGA	GCCAACTTCCTCCAAGAGC
Axl	TGGCTGTGAAGACGATGAAG	GTCAAATTCCTTCATGCAGACC
Itgam	GCCTGGATTATAAGGATGTC	TTGAAAAGCTAATCCAACCC
Il-1β	CTAAACAGATGAAGTGCTCC	GGTCATTCTCCTGGAAGG
Ccl22	GTGGTGTTGCTAACCTTC	GGCTCAGCTTATTGAGAATC
Il-10	GCCTTTAATAAGCTCCAAGAG	ATCTTCATTGTCATGTAGGC

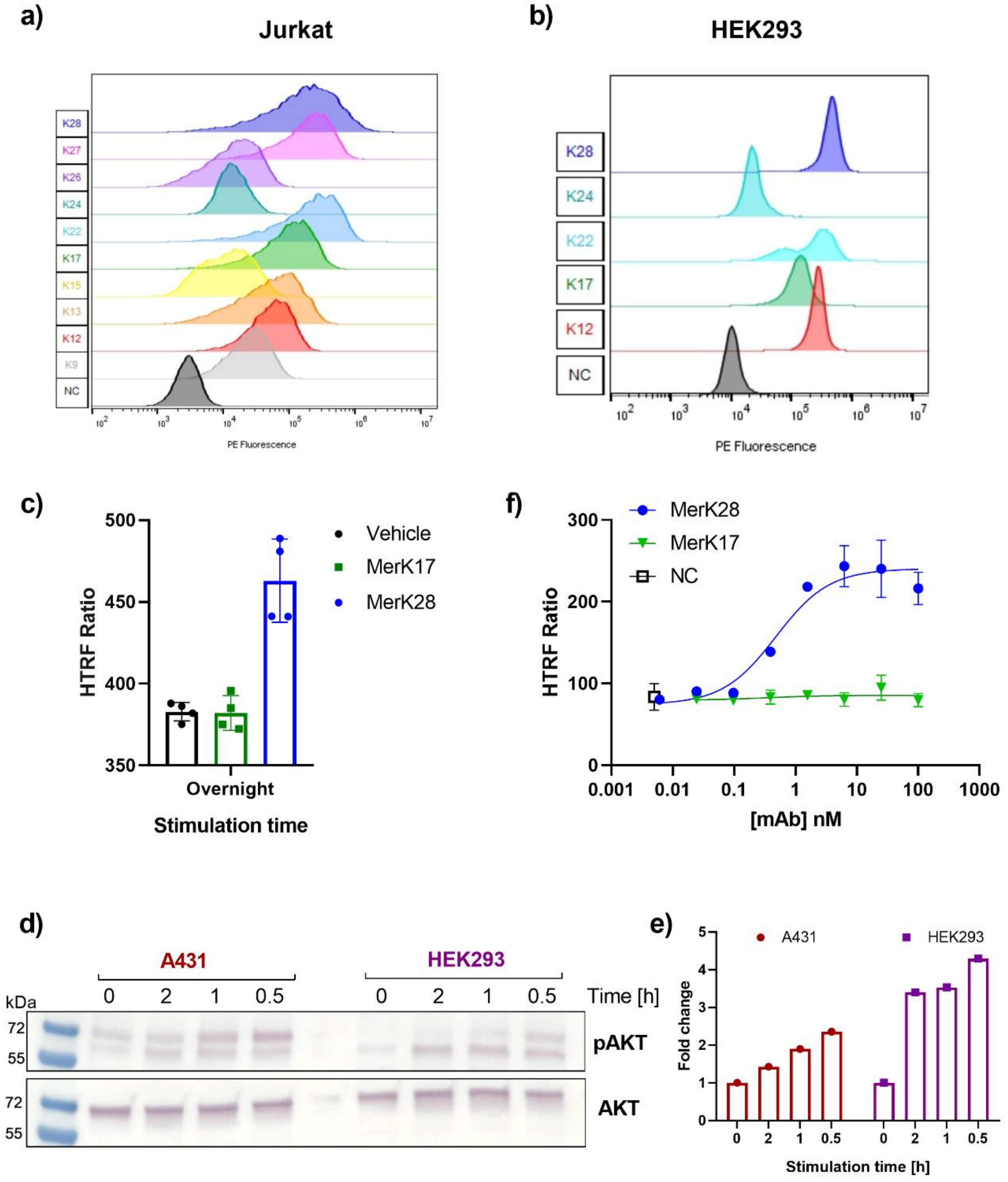

Figure S1: Characterisation of all MerTK antibodies and signalling activation of MerK17 and MerK28. Cell staining with 250 nM anti-MerTK antibodies of a) Jurkat or b) HEK293. Bound antibodies were detected with an anti-human Fc PE-conjugate. Colour-coding represents the different clone numbers. C) Overnight stimulation of A549 cells with either 50 µg/ml MerK17 or MerK28 or a vehicle (PBS). d) Stimulation with 50 µg/ml MerK28 on either A431 or HEK293 cells for the indicated time points. The pAKT levels were normalized to total AKT and set relative to the negative control to determine the fold change induction of pAKT after stimulation (e). f) 30 min incubation of A549 cells with the indicated antibody concentration. HTRF Ratio represents the ratio of donor and acceptor values are suggested by the manufacturer.

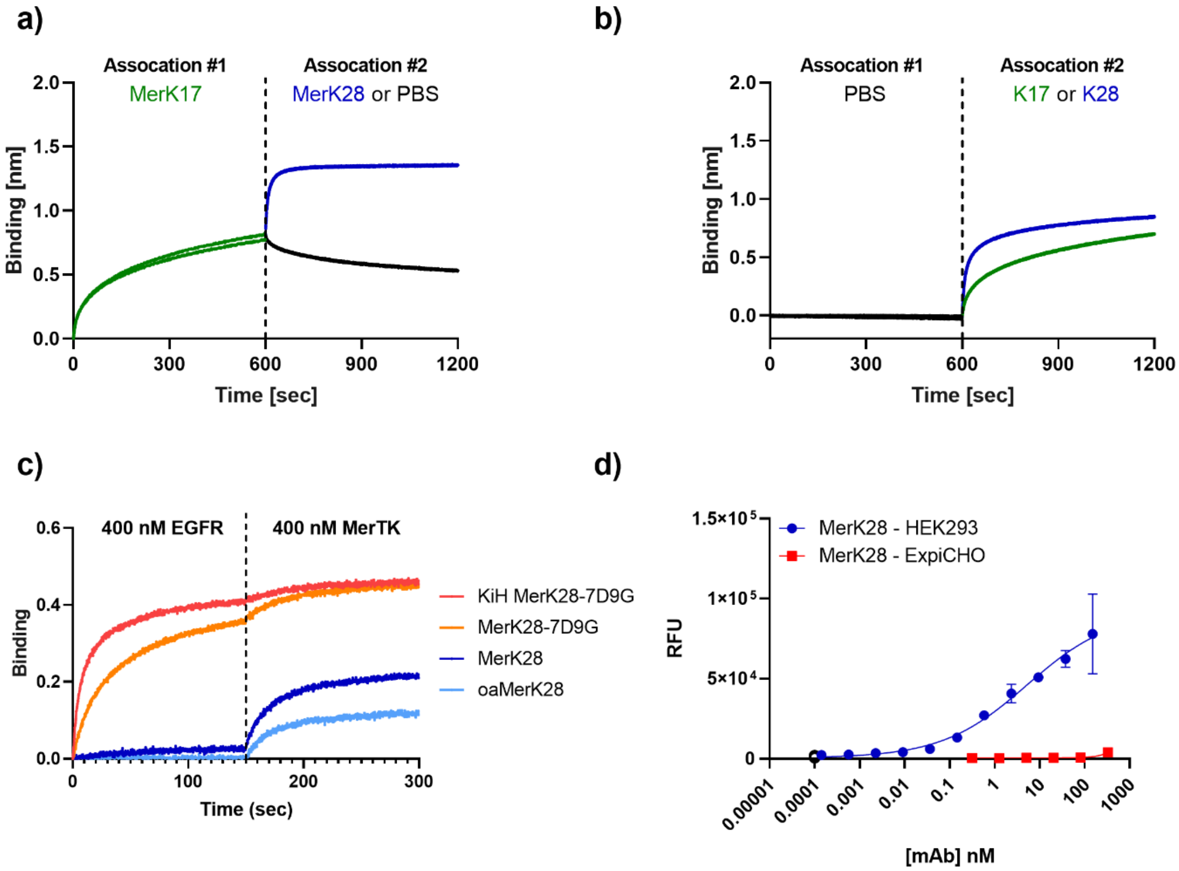

Figure S2: Epitope binning and characterisation of bispecific antibodies. a) Epitope binning by BLI. Association of MerK17 (green) followed by either MerK28 (blue) or PBS (black). b) Competition assay of MerK17 for the ligand binding site of Pros1 *via* BLI. MerK17 was loaded onto AHC biosensors and sequentially associated to either 500 nM MerTK (light blue) or 500 nM MerTK pre-incubated with equimolar Pros1 (pink). c) Simultaneous binding of human MerTK and EGFR via BLI. Antibodies were loaded onto AHC biosensors and then associated first to EGFR and then to MerTK over 300 seconds. d) Cellular binding of MerK28 on either HEK293 (blue) or ExpiCHO cells as a negative control shown in red.

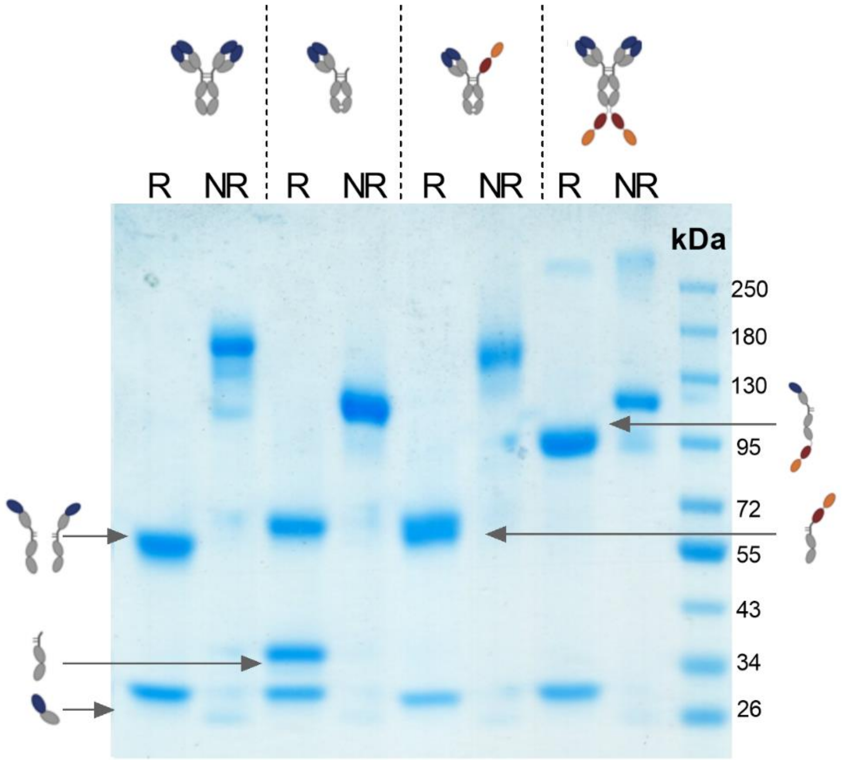

Figure S3: SDS-PAGE gel analysis of all variants in reducing and non-reducing conditions, respectively. The reduced fragments of the variants are indicated with arrows at their expected sizes. Abbreviations: R – reduced, NR – non-reducing, kDa- kilodaltons.

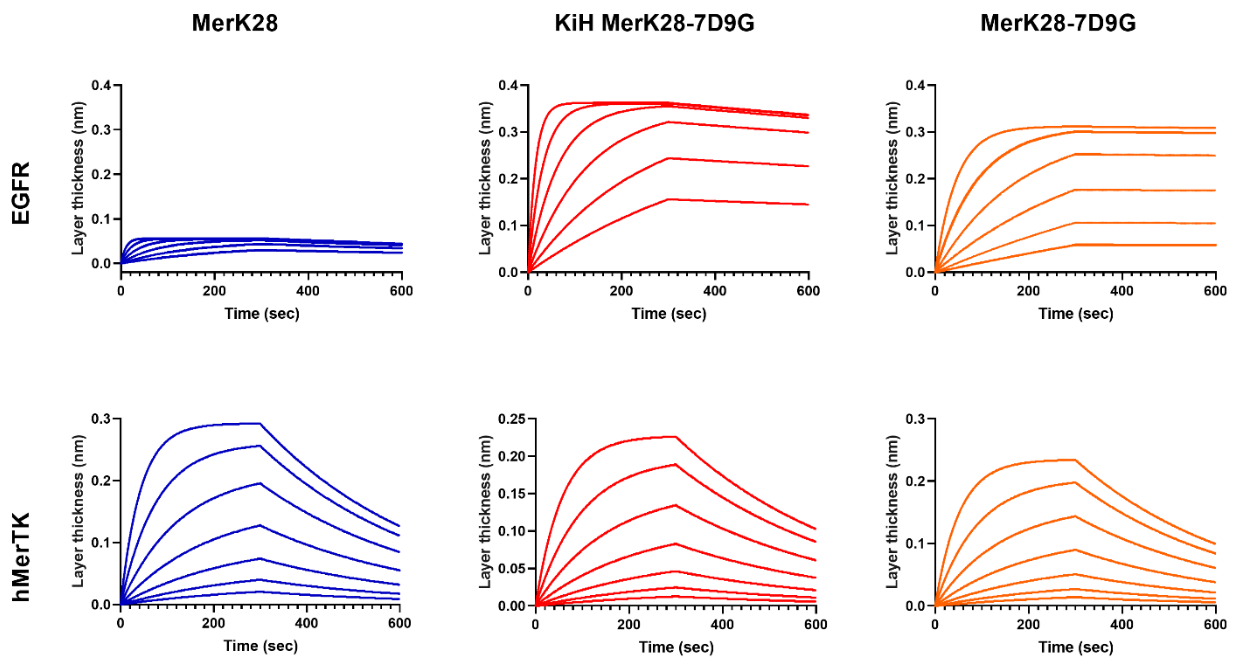

Figure S4: Kinetics Determination. The indicated antibodies were immobilised onto anti-human Fc capture (AHC) biosensors and associated to different target antigen concentrations. For both hMerTK and EGFR, a range of 200 – 0 nM were measured using a 2-fold serial dilution.

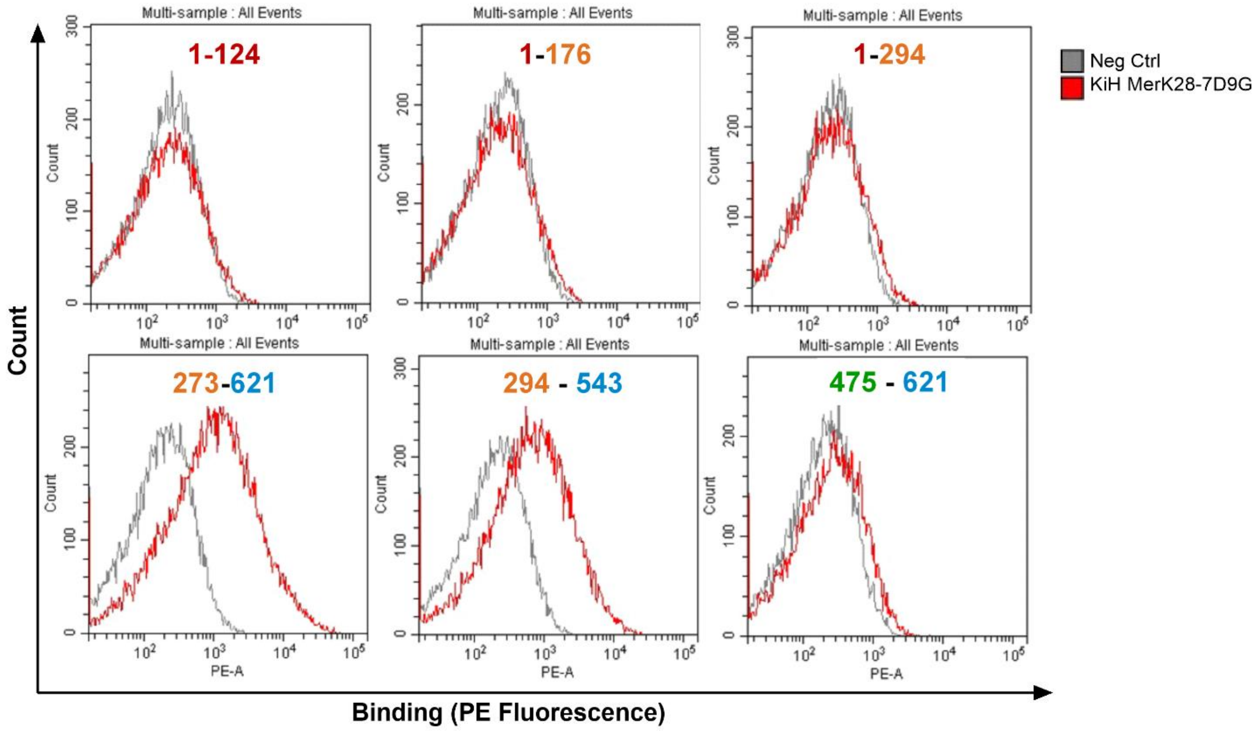

Figure S5: EGFR domain mapping on YSD. Numbers represent the amino acids of the extracellular domain of EGFR, as displayed on yeast cells. The grey histograms present the negative control staining with only secondary Ab, while the red represents KiH MerK28-7D9G. EGFR domain mapping colour-coding: red – domain I, orange – domain II, green – domain III, and blue – domain IV. Yeast cells were stained with 100 nM of the indicated mAbs followed by anti-human Fc-PE-conjugate staining (1:75 dilution). 10'000 events were measured for each.

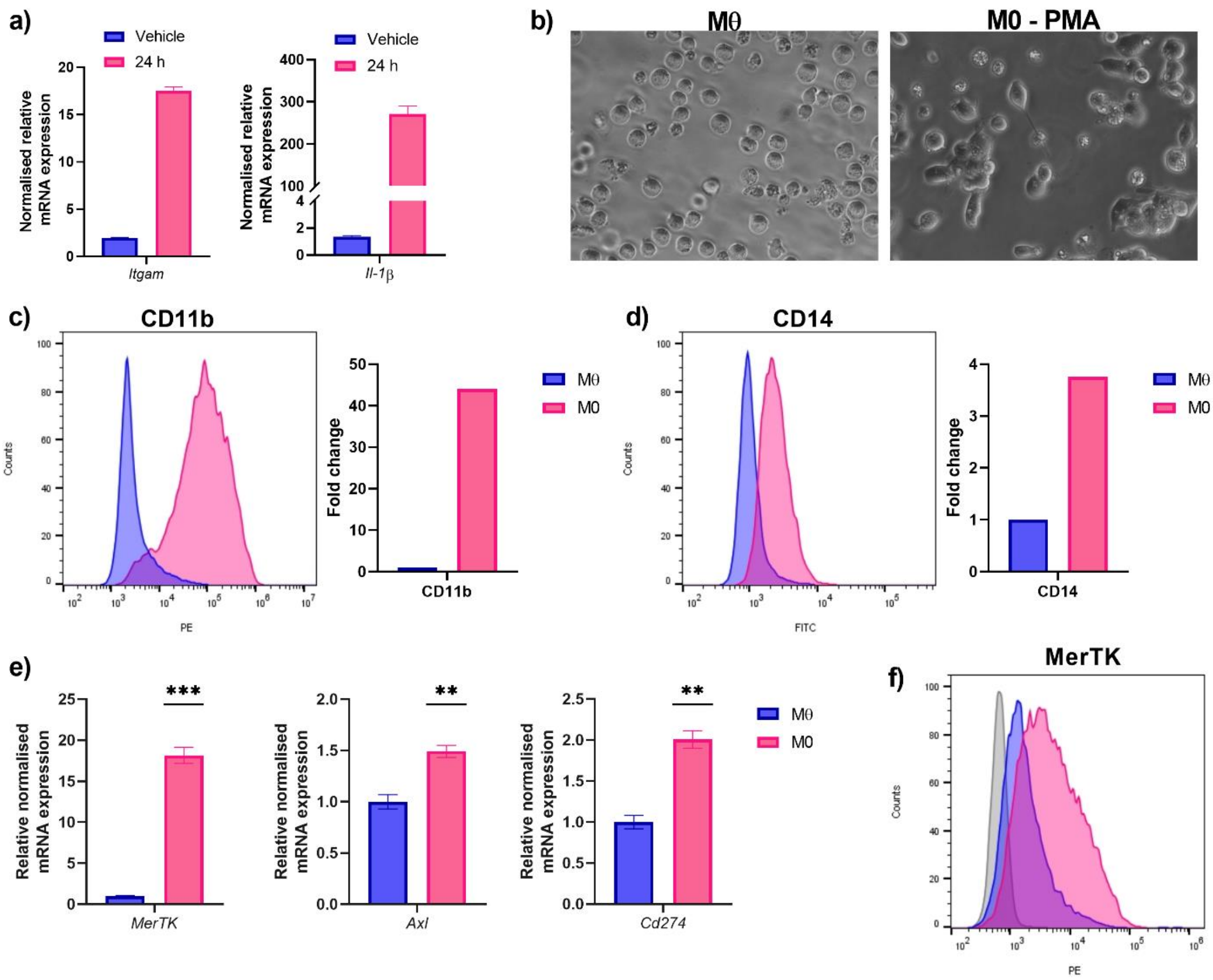

Figure S6: Differentiation of THP-1 cells after PMA incubation. a) Gene expression analysis of unstimulated (blue) or PMA-stimulated THP-1 cells (pink). *Itgam* and *Il-1b* mRNA levels were determined and set relative to the unstimulated control, normalised to internal housekeeping genes. b) Brightfield microscopy images of THP-1 cells before and after PMA incubation. c) Flow cytometric analysis of cell-surface CD11b measured with an anti-CD11b PE-conjugate. d) Cell surface expression of CD14 measuring using an anti-CD14 FITC-conjugate. Fold change was determined for c) and d) by calculating the mean fluorescence and setting the data relative to the Mθ control. e) Relative normalised mRNA expression of *MerTK*, *Axl* and *Cd274*. f) MerTK cell surface levels as determined by staining with 50 μg/ml MerK28 and an anti-human Fc PE-conjugate. The grey histogram represents the secondary antibody only, while the blue and pink represent Mθ and M0 THP-1s, respectively. Error bars for RT-qPCR represent SEM of triplicate measurements, while flow cytometric analysis is representative of a single experiment while it was repeated at least three times.

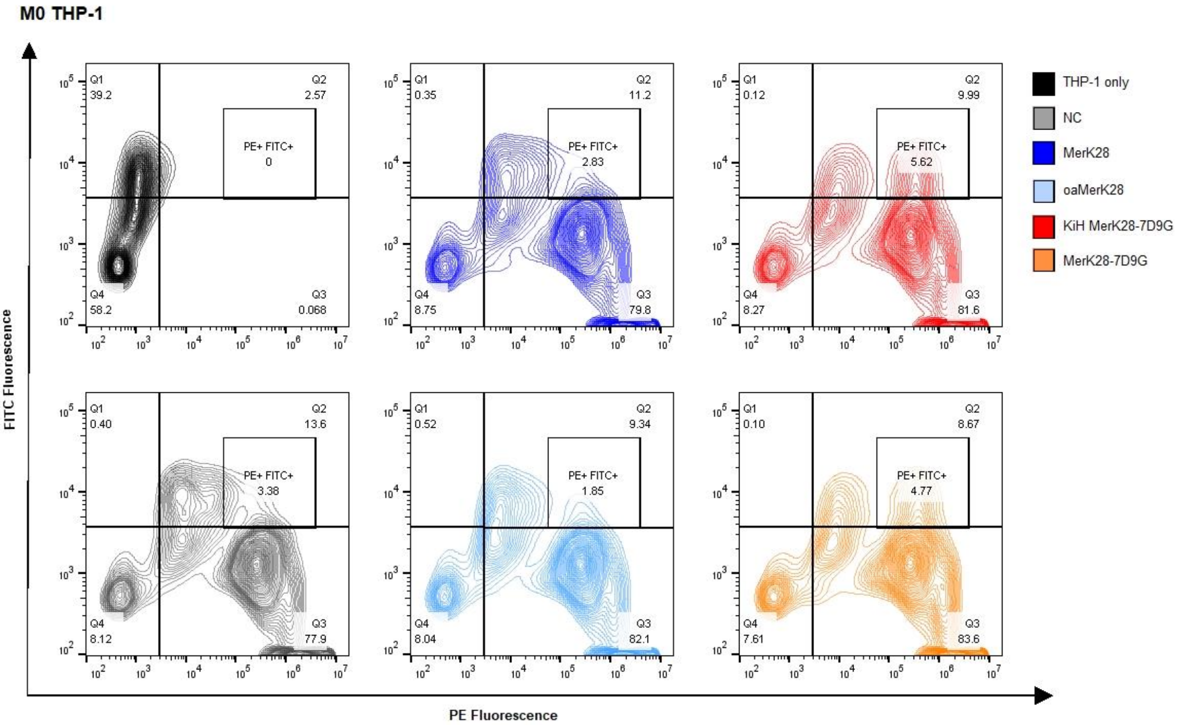

Figure S7: Contour plots of M0-like THP-1 cells after co-incubation with A431 cells. A431 cells were stained with Calcein-AM red-orange and measured using the PE channel (x-axis), whereas THP-1 cells were gated after staining with an anti-CD14-FITC antibody (y-axis). To determine phagocytosis, the double-positive PE+/FITC+ events in the upper right quadrant Q2 were analysed and gated further. Signal in Q3 only represents possible unspecific A431 cells sticking to the THP-1 cells. Colour-coding is indicated in the legend. The negative control (NC, gray) represents THP-1 and A431 cells without any antibody treatment. Visualisation was performed using FlowJo v10 software.

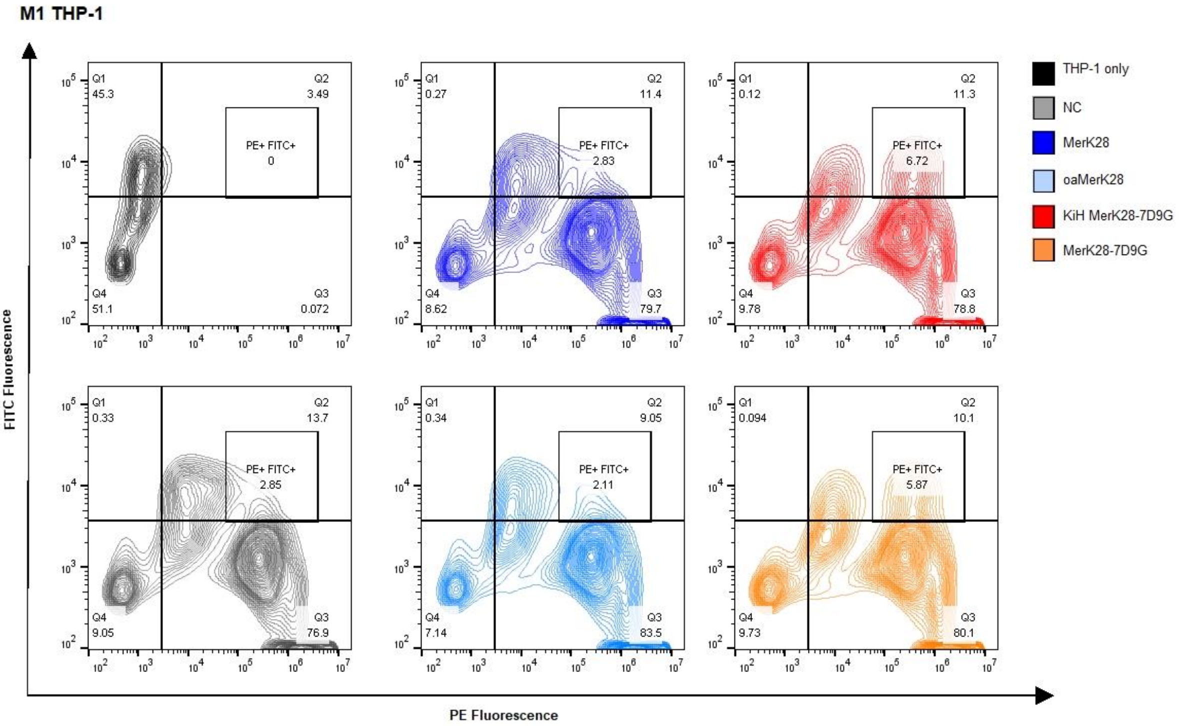

Figure S8: Contour plots of M1-like THP-1 cells after co-incubation with A431 cells. A431 cells were stained with Calcein-AM red-orange and measured using the PE channel (x-axis), whereas THP-1 cells were gated after staining with an anti-CD14-FITC antibody (y-axis). To determine phagocytosis, the double-positive PE+/FITC+ events in the upper right quadrant Q2 were analysed and gated further. Signal in Q3 only represents possible unspecific A431 cells sticking to the THP-1 cells. Colour-coding is indicated in the legend. The negative control (NC, gray) represents THP-1 and A431 cells without any antibody treatment. Visualisation was performed using FlowJo v10 software.

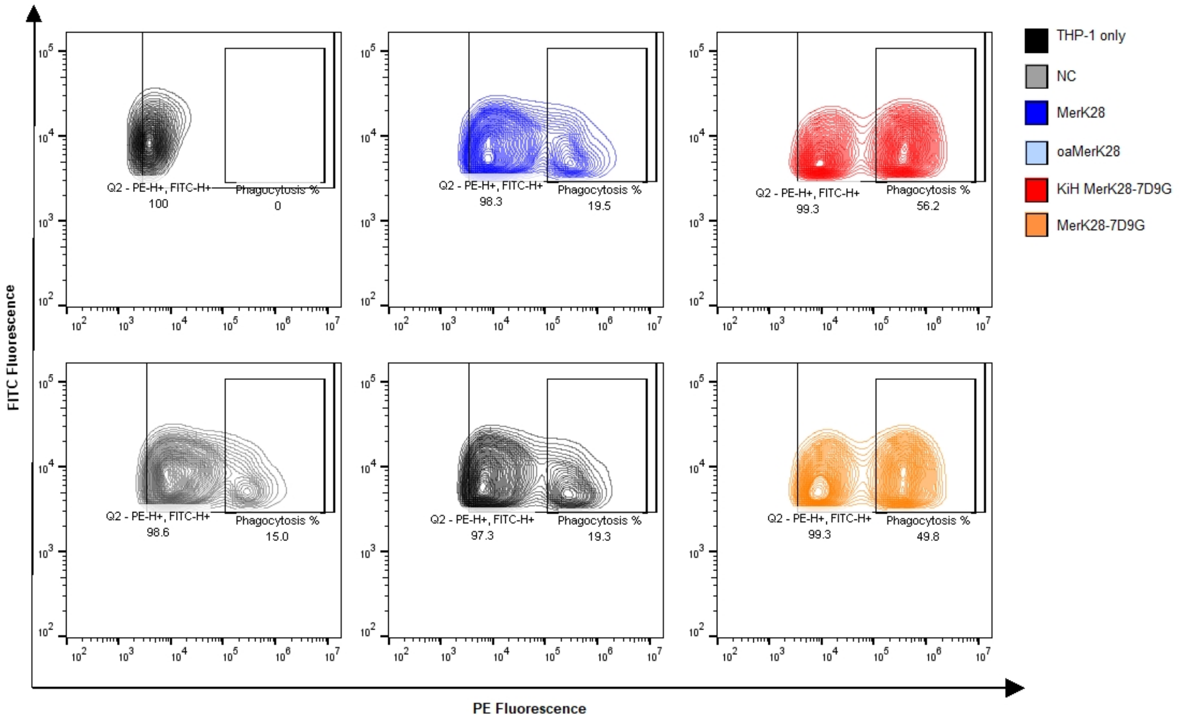

Figure S9: Phagocytosis rate % determination by gating the PE++ events as exemplified by M1-like THP-1 cells. From the gated PE+/FITC+ cells in Figure S8, the co-incubation of cells were gated further to ensure the THP-1 cells had taken up the A431 cells and were not bound lightly to their surface ("phagocytosis %"). A431 cells were stained with Calcein-AM red-orange and measured using the PE channel (x-axis), whereas THP-1 cells were gated after staining with an anti-CD14-FITC antibody (y-axis). Colour-coding is indicated in the legend. The negative control (NC, gray) represents THP-1 and A431 cells without any antibody treatment. Visualisation was performed using FlowJo v10 software.
